# Traumatic Facial Nerve Palsy

**DOI:** 10.5811/cpcem.2017.5.32970

**Published:** 2017-09-29

**Authors:** Brenna Derksen, Sherri Rudinsky

**Affiliations:** * University of California San Diego, Department of Emergency Medicine, San Diego, California; † Rady Children’s Hospital, Department of Emergency Medicine, San Diego, California

## CASE PRESENTATION

**Image 1 AB:**
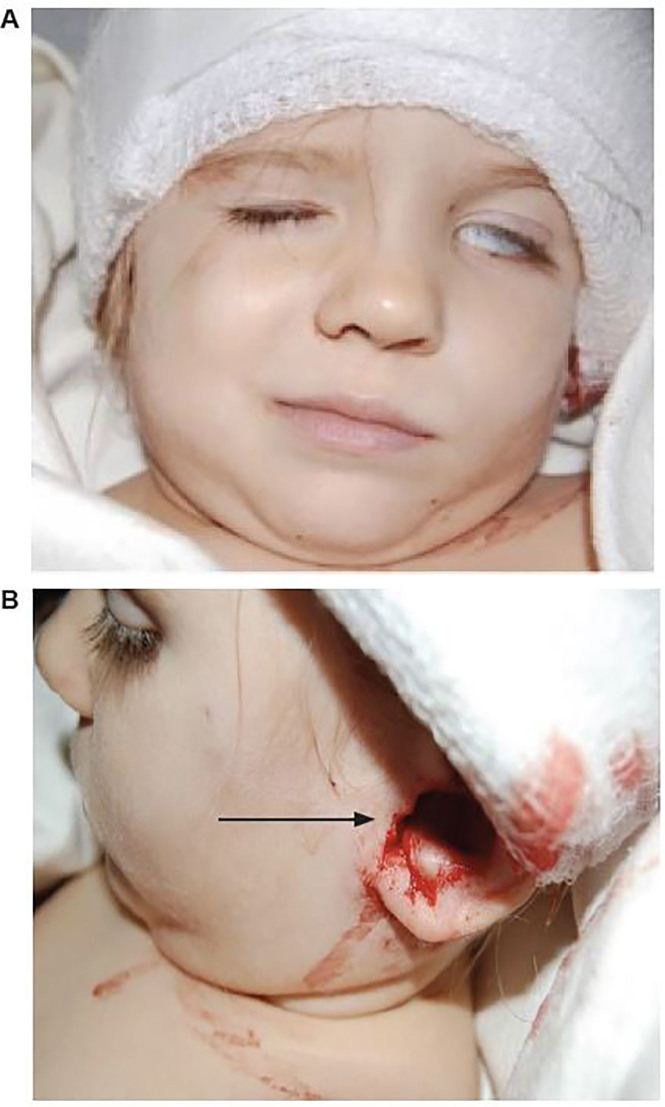
A) Left facial acial nerve palsy and B) stellate laceration with puncture wound to left ear (arrow).

**Image 2:**
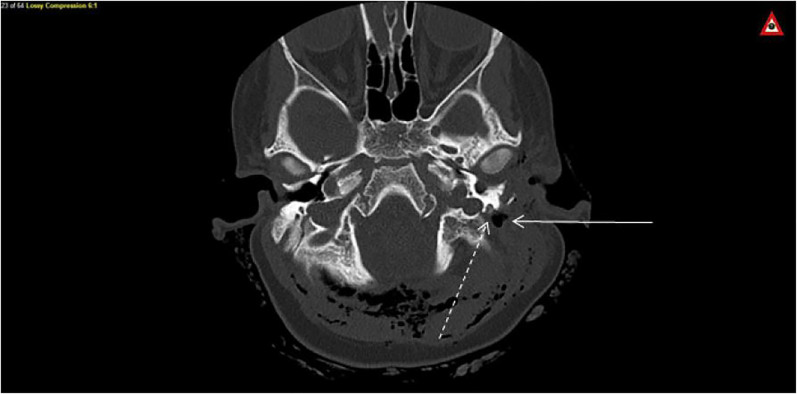
Computed tomography demonstrating soft tissue swelling (solid arrow) abutting the hyperdense area of the facial nerve, sounded by bone (dashed arrow)

A two-year-old female presented to the emergency department with facial lacerations after an attack by the family canine (Boxer breed). The exam revealed a stellate laceration on the cartilage of her left pinna, blood in the ear canal, left-sided facial droop, and inability to close her left eye (Image 1). Computerized tomography (CT) and magnetic resonance imaging (MRI) showed avulsion fractures of the left temporal bone, soft tissue emphysema, and an edematous, hyperemic facial nerve (Image 2). The patient was admitted for intravenous antibiotics, operative repair of her temporal bone fractures, and decompression of cranial nerve (CN) VII via mastoidectomy. She was discharged on a steroid taper with minimal improvement in her facial palsy.

## DISCUSSION

Temporal bone fractures can result in facial nerve paresis/paralysis if CN VII is involved.^1^ Facial and skull fractures associated with dog bites in children may result in significant intracranial injuries often requiring complex surgical repair.^2^,^3^ Given the thinness of cranial bones in children and high pressures associated with dog bites (200–450 psi), crush injuries and puncture wounds from canine teeth can occur despite minimal skin defects.^3^,^4^ Delayed diagnosis of injuries is not uncommon, resulting in significant morbidity.^3^,^4^ CT imaging should be considered early, and MR angiography should be obtained in cases of penetrating trauma.^1^,^2^–^4^ Patients with intracranial injury, neurologic deficits, or temporal bone disruption may benefit from early surgical intervention.^1^,^2^ Additional management considerations include operative debridement, parenteral antibiotics, corticosteroids, and prophylactic corneal care.^1^–^4^

**CPC-EM Capsule**

What do we already know about this clinical entity?

*The thin temporal bones of children are susceptible to* *high-pressure injuries associated with dog bites, often resulting in damage to important structures such as cranial nerve VII.*

What is the major impact of the image(s)?

This image demonstrates that high-pressure injuries from canine teeth can result in significant intracranial pathology despite minimal skin defects.

How might this improve emergency medicine practice?

*This image highlights the importance of imaging in cases of penetrating facial trauma from dog bites to avoid delayed diagnosis and facilitate early intervention.*
